# Self-Leadership Among Healthcare Workers: A Mediator for the Effects of Job Autonomy on Work Engagement and Health

**DOI:** 10.3389/fpsyg.2020.01420

**Published:** 2020-07-16

**Authors:** Pauline van Dorssen-Boog, Jeroen de Jong, Monique Veld, Tinka Van Vuuren

**Affiliations:** ^1^Faculty of Management, Open University of the Netherlands, Heerlen, Netherlands; ^2^Intrinzis, Delft, Netherlands; ^3^School of Management, Institute for Management Research, Radboud University, Nijmegen, Netherlands; ^4^BrabantZorg, Oss, Netherlands; ^5^Loyalis Knowledge & Consult, Heerlen, Netherlands

**Keywords:** job autonomy, self-leadership, work engagement, health, healthcare workers

## Abstract

Due to the high workload, working within the healthcare industry can be quite demanding. This often results in high rates of absenteeism, unfulfilled vacancies, and voluntary turnover among healthcare workers. We expect that job autonomy is an important resource for work engagement and health of healthcare workers because it satisfies the basic need for autonomy. However, we propose that this relationship between job autonomy and work engagement and health can be explained by self-leadership. Self-leading individuals take initiative and responsibility and are assumed to use self-influencing strategies (e.g., goal setting, self-observation, creating natural rewards) as a way to improve motivation and general well-being. Employees from two healthcare organizations (*N* = 224 and *N* = 113) completed a questionnaire containing measures of job autonomy, work engagement, general health, and self-leadership. The hypothesized model was tested using a series of regressions, and the results confirmed the indirect relationships between job autonomy and work engagement and general health, respectively, through natural rewards strategies. The behavior-focused and cognitive self-leadership strategies were, as mediator, marginally significant: positively for work engagement and negatively for general health. Self-leadership behavior was not related with work engagement and general health. Implications of the findings for theory and practice on healthy healthcare workers are discussed.

## Introduction

Working within healthcare is often valued as meaningful, energizing, and engaging as this type of work is expected to generate feelings of meaningfulness and joy throughout a career (De Cooman et al., 2008; Toode et al., 2011). However, healthcare workers around the world also report that their work is demanding, stressful, and dissatisfying, resulting in high rates of absenteeism and premature exit from this specific labor market (Garrosa et al., 2008; Estryn-Behar et al., 2010; Hayes et al., 2012).

Drawing on the job demand control model (Karasek, 1979), it has been repeatedly suggested that reduced well-being among healthcare workers is a result of the interaction between the high workload and low job control of the jobs within the healthcare industry (e.g., Laschinger et al., 2001). Therefore, scholars suggest that increasing job autonomy is one of the job design measures that should be taken in order to improve the motivation and health of healthcare workers (Widerszal-Bazyl et al., 2003; Cicolini et al., 2014). Job autonomy refers to the amount of freedom and independence within a job as well as the discretion of the individual in scheduling the work and determining the procedures (Hackman and Oldman, 1976). Self-determination theory (SDT, Deci et al., 2017) explains that people have a basic psychological need for autonomy, which they want to satisfy. Through satisfaction of this need, people are allowed to make their own choices and bring activities in line with their own values and interests, leading to intrinsic motivation, vitality, personal growth, and general health (Ryan and Deci, 2000, 2008). According to Hall (1968), job autonomy enables dedicated professionals, such as nurses and social workers, to self-regulate their job tasks in a responsible way (Hall, 1968). The basic assumption is that, if employees are well educated for their profession, they are assumed to be willing and able to autonomously regulate their own job tasks responsibly. They are able to solve daily problems and proactively ask feedback from colleagues if necessary. Therefore, the facilitation of job autonomy is needed for being able to professionally do one’s job as healthcare professional (Hall, 1968).

However, despite the growing support for job autonomy as an important job design measure for healthcare professionals, employees seem to differ in the effectiveness of the interaction between job control and job demands (Presseau et al., 2014). If healthcare workers are confronted with high job demands while being facilitated with job autonomy, they need to possess competencies for self-control and self-determination (Wagner et al., 2010). In other words, we propose that they need to have competencies for self-leadership.

Self-leadership theory assumes that people can autonomously direct and motivate themselves (Manz, 1986, 2015). Self-leadership refers to “a comprehensive self-influence perspective that concerns leading oneself toward performance of naturally motivating tasks as well as managing oneself to do work that must be done but is not naturally motivating” (Manz, 1986, p. 589). It is assumed that self-leadership can play a distinctive role for healthcare professionals working in high-strain jobs (Lovelace et al., 2007). Through practicing self-leadership, people might be able to positively influence their motivation and health even if their job autonomy is low (Lovelace et al., 2007; Stewart et al., 2019). Within the healthcare literature, there is growing evidence for the potential benefits of self-leadership for the well-being and performance of healthcare professionals (e.g., Jooste and Cairns, 2014; Kayral and Dülger, 2019; Kim and Kim, 2019). Still, self-leadership theory assumes that an autonomy-supportive work context is beneficial for the self-leadership of employees as they are encouraged to actually take up responsibility for their job and increasingly use cognitive and behavioral self-influencing strategies in order to optimize their own motivation and performance (Stewart et al., 2019).

**FIGURE 1:**
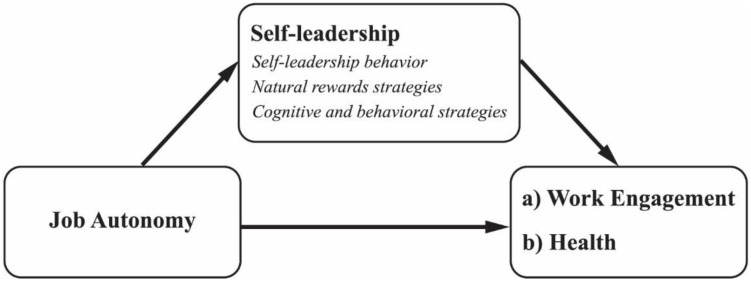
Conceptual model.

In the present study, we draw on SDT (Deci et al., 2017; Ryan and Deci, 2017) to explain why self-leadership is a critical mediator in the relationships between job autonomy and work engagement and the health of healthcare professionals. We propose that, if healthcare professionals are facilitated with job autonomy, this directly associates with work engagement and health and also indirectly through the practice of self-leadership (Lovelace et al., 2007; Stewart et al., 2011; Figure 1). The assumptions are tested with a sample of healthcare professionals from two different Dutch organizations: a nursing home and an organization for disability- and psychiatric care.

With this study, we aim to contribute to the existing literature in several ways. First, we integrate insights from SDT in the motivational process (Gagné and Deci, 2005; Deci et al., 2017) with self-leadership theory (Manz, 2015; Stewart et al., 2019). SDT proposes that people are inherently intrinsically motivated, which can be thwarted if the basic psychological need for autonomy is not satisfied, for instance, by a controlling work context. However, self-leadership theory assumes that people are not merely a result of controlling external regulation as they can self-influence their motivation and behavior, including their health (Lovelace et al., 2007). In the present study, we test whether self-leadership explains the proposed relationship between job autonomy and work engagement and health, respectively.

Second, we contribute to the self-leadership literature as we have separated three different aspects of the self-leadership process: actual self-leadership behavior, natural rewards strategies, and the use of behavioral and cognitive strategies. Self-leadership studies often focus on one dimension of self-leadership (e.g., Yun et al., 2006; Zeijen et al., 2018), resulting in limited insight into the self-leadership process. The present study includes both the self-influencing strategies (i.e., natural rewards strategies and cognitive and behavioral strategies) and the actual self-leadership behavior as these might have different relationships with job autonomy and the outcomes on work engagement and health.

Third, the present study is specifically focused on healthcare professionals. Healthcare literature assumes that both organizational interventions and individual coping strategies (McVicar, 2003) are important considerations to investigate optimal work conditions for these professionals. The present study is among the first to test the influence of both job autonomy and self-leadership on the work engagement and health of healthcare professionals.

## Theoretical Background

### The Role of Autonomy in Work Engagement and Health

According to SDT, autonomy plays an important role in the motivational process of employees. Autonomy refers to the regulation by the self (Ryan and Deci, 2006). It involves acting with a sense of volition and having the experience of choice (Gagné and Deci, 2005, p. 333). By referring to the philosopher Dworkin (1988), SDT theorizes that autonomy is represented by the full endorsement of one’s actions at the highest level of reflection (Gagné and Deci, 2005).

SDT assumes that people have a basic psychological need for autonomy, which they want to satisfy (Deci and Ryan, 2000). The psychological experience of autonomy allows people to freely choose their activities. If motivation is based on autonomy, it is more integrated with personal goals, values, and interests and ultimately based on intrinsic motivation (Gagné and Deci, 2005). Intrinsic motivation is recognized by the implicit interest and enjoyment for a task or activity itself. Intrinsic motivation is fully volitional and is associated with increased levels of vitality, energy, health, and personal growth (Ryan and Deci, 2008; Deci et al., 2017).

In contrast, if activities are not based on autonomous choices, they require external behavior regulation. The enactment depends upon the perception of the contingency between the behavior and another desired consequence. For instance, one acts to avoid negative feedback or to receive specific tangible rewards. If motivation is externally regulated, it is based on control (Ryan and Deci, 2000; Gagné and Deci, 2005). Activities are done because they *must* be done, which triggers a sense of pressure and strain. Therefore, extrinsic or controlled motivation is associated with increased levels of stress and with the impairment of health (Ryan and Deci, 2008; Van den Broeck et al., 2011; Weinstein and Ryan, 2011).

SDT assumes that, if the job context is highly controlling, meaning that the level of freedom and independence in a job is low, this can reduce the intrinsic motivation and health because the basic need for autonomy is thwarted (Deci et al., 1999; Gagné and Deci, 2005). If professions, such as healthcare workers, are not free to responsibly determine their own way of working, their behavioral intentions are regulated by external control. For instance, if healthcare institutions try to regulate employees’ behaviors through an abundance of procedures and feedback systems, employees might be more motivated to achieve these external goals than to deliver the care they want to deliver to their clients. More specifically, employees might act in order to prevent themselves from negative feedback from the manager or in order to receive compliments by managers as a way to boost their self-esteem. Work behavior tends to be based on what one *must* do (controlled motivation) instead of what one is *willing* to do (autonomous motivation). It is assumed that, even if nurses are originally intrinsically motivated for a job task, the implementation of external control can easily distract them, leading to an increased strain and reduced intrinsic motivation (Gagné and Deci, 2005). In contrast, if employees can define their own way of working more freely, they are assumed to value the work more for its inherent joy and meaningfulness.

Intrinsic work motivation is theorized to be represented by the concept of work engagement (Salanova and Schaufeli, 2008). Work engagement refers to a positive, fulfilling, work-related state of mind, which is characterized by dedication (i.e., strong involvement, enthusiasm, pride, and experience of significance), vigor (i.e., high levels of energy and mental resilience), and absorption (full concentration and difficulties with detaching oneself from work) (Schaufeli et al., 2006). Work engagement is assumed to be an indicator of the general autonomous and intrinsic motivation at work (Salanova and Schaufeli, 2008; Van Beek et al., 2012). Where intrinsic motivation can be specifically focused on one job task, work engagement is not specifically focused on a momentary state, object, event, individual, or behavior. It reflects a more persistent and pervasive affective-cognitive state (Schaufeli et al., 2006). Engaged workers work because they genuinely *want* to work (Salanova and Schaufeli, 2008). It is assumed that work engagement predicts positive organizational outcomes, such as customer satisfaction, because engaged workers are willing to go the extra mile (Bakker et al., 2014).

There is abundant evidence available to support that job autonomy is an important resource for work engagement and health (Bakker and Demerouti, 2007; Van den Broeck et al., 2008; Bakker et al., 2014). Within healthcare, job autonomy seems to be a predicting factor for work engagement and mental and physical health of healthcare workers (Toode et al., 2011). For instance, evidence is found that home care nurses report significantly more work engagement and lower levels of burnout when facilitated with autonomy (Vander Elst et al., 2016). Furthermore, it was proven that job autonomy is an important resource for nurses working within the hospital setting as it contributes to their work engagement (Vera et al., 2015). And Madathil et al. (2014) found in a sample of psychiatric nurses that they report lower levels of burnout if they are facilitated with job autonomy (Madathil et al., 2014).

Therefore, we hypothesize the following:

Hypothesis 1: Job autonomy is positively associated with (a) work engagement and (b) general health of healthcare workers.

### Self-Leadership: The Actual Autonomous Functioning

Although SDT has the premise that satisfaction of the need for autonomy plays an important role in work engagement and health (Ryan and Deci, 2008; Van den Broeck et al., 2008; Deci et al., 2017), it does not describe strategies on *how* people can autonomously control the motivational process (Bakker and Van Woerkom, 2017). In fact, SDT assumes that the satisfaction of the need for autonomy inherently leads to autonomous functioning and intrinsic motivation (Ryan and Deci, 2017).

However, self-leadership theory describes the process of self-influence with the aim to optimize motivation and general performance (Neck and Houghton, 2006). Self-leaders strive to regulate their cognition and behavior in such a way that work and life become more aligned with personal goals, needs, and interests and, therefore, become more valuable, meaningful, and enjoyable (Manz, 1986, 2015). People who take the lead act on the basis of authentic or autonomous choices (Yun et al., 2006; Manz, 2015; Stewart et al., 2019). A self-leader is assumed to autonomously define *what* to do (standards and objectives), *why* to do things (strategy), and *how* to do things (methodology) while being less dependent on contextual control systems (Manz, 1986; Stewart et al., 2011). True self-leadership represents autonomous functioning as one can fully endorse personal activities and act on a basis of higher order reflections (Manz, 2015).

So as to effectively function in an autonomous way, self-leaders are assumed to use specific behavioral and cognitive self-influencing strategies with the aim to optimize motivation, well-being, and performance (Manz, 1986, 2015; Neck and Houghton, 2006). These strategies are classified in three basic categories, which are behavior-focused strategies, constructive thought pattern strategies, and natural reward strategies. *Behavior-focused strategies* (e.g., self-observation, self-goal setting) can be used for self-motivation and self-direction in case tasks are difficult, boring, or otherwise challenging but still need to be done. They are especially helpful in tasks and goals that are based on extrinsic motivation (Manz, 1986; Houghton and Neck, 2002; Neck and Houghton, 2006). *Constructive thought pattern strategies* (e.g., mental imagery, positive self-talk, and evaluation of thoughts, and assumptions) aim to mentally motivate oneself to achieve job tasks and manage functional patterns of habitual thinking (Neck and Manz, 1992, 1996). They generally focus on opportunities rather than threats and can help to reduce negative thoughts about a job task or situation and to construct more positive and helpful thoughts (Neck and Houghton, 2006). And finally, *natural reward strategies* refer to both behavioral and cognitive strategies, aimed at fostering positive affect and intrinsic motivation (Neck and Houghton, 2006). Natural rewards can be achieved by actively creating more attractive job conditions. Aside from that, one can also cognitively increase natural rewards by changing the mental focus from unpleasant aspects within a task to pleasant, naturally rewarding aspects of the task (Neck and Houghton, 2006).

### Job Autonomy and Self-Leadership

Several scholars have theorized that self-leadership can be facilitated by highly autonomous job contexts (Alves et al., 2006; Stewart et al., 2019). It is assumed that, if employees are given substantial freedom in their jobs, employees tend to more autonomously define what to do, why to do things, and how to do things while being less dependent on instructions by external leaders (Manz, 1986; Stewart et al., 2011). Moreover, as a result of job autonomy, employees are more dependent on their own cognitive and behavioral self-influencing strategies as the external directions and cues are missing (Alves et al., 2006; Müller and Niessen, 2019). Indeed, Müller and Niessen (2019), in a study among teleworkers, found that on days when employees work from home, they make significantly more use of self-leadership strategies (self-reward, self-goal setting, visualization of successful performance, and evaluation of beliefs and assumptions), which was explained by the perceived job autonomy. Furthermore, some studies found evidence for the moderating influence of job autonomy on the association between self-leadership and job satisfaction (Roberts and Foti, 1998; Ho and Nesbit, 2014) and performance, respectively (Ho and Nesbit, 2014). Moreover, Hornung and Rousseau (2007) found that job autonomy can have long-term effects on personal initiative of hospital workers over a time period of 18 months while the reverse effect measured in the same period was not significant.

### The Effects of Self-Leadership on Work Engagement and Health

Self-leadership theory is based on the early work by Deci (1975) as it acknowledges the difference between extrinsic and intrinsic motivation for behavioral outcomes and well-being. True self-leadership is based on autonomous choices and intrinsic motivation (Manz, 1986, 2015). However, self-leadership theory recognizes that a job always has tasks that are not naturally motivating but simply need to be done. For these types of tasks, self-leaders can use the self-management strategies (Manz, 1986; Stewart et al., 2011, 2019). Self-management refers to the self-influencing process aiming to meet externally set standards and objectives. For instance, when an employee needs to follow strict regulations within a job task, this procedure is not autonomously chosen and, hence, externally determined. Still, the individual can self-manage motivation and behavior by using cognitive and behavioral self-influencing strategies. The use of behavior-focused strategies, such as self-observation, goal-setting, and tangible self-rewards can function as powerful motivators for actual performance. And constructive thought pattern strategies and natural rewards strategies are helpful for making boring, difficult, or otherwise challenging job tasks more naturally rewarding or, at least, more meaningful (Neck and Houghton, 2006).

Indeed, evidence is growing for the influence of self-leadership on outcomes related to work engagement. Breevaart et al. (2016) find support for the idea that actual autonomous self-leadership behavior (i.e., taking responsibility and initiative in an independent way) is associated with work engagement. In a weekly diary study, it was found that, in weeks in which employees show more self-leadership, they also report higher rates of work engagement (Breevaart et al., 2016). Furthermore, Breevaart et al. (2014) find, in a daily diary study among maternity nurses, that behavior-focused self-leadership strategies (self-goal setting, self-observation, and self-cueing) had positive effects on work engagement through the mediating effect of the specific job resources “feedback” and “developmental opportunities” (Breevaart et al., 2014). There is also evidence for the influence of cognitive self-leadership strategies on outcomes related to well-being and job satisfaction as it was confirmed that this relationship is negatively mediated by dysfunctional thought processes (Houghton and Jinkerson, 2007). Furthermore, natural rewards strategies are assumed to play a central role in the motivational process as they are specifically aimed to improve intrinsic motivation (Furtner et al., 2015). Furtner et al. (2012) investigated, with an intervention study among a group of psychology students, which self-leadership strategies were perceived as most beneficial for improving their motivation and performance for their studies. It was found that the students most appreciated the natural rewards strategies as these were helpful to increase their intrinsic motivation during their studies (Furtner et al., 2012). Furthermore, evidence finds that natural rewards strategies are negatively associated with fear of failure (Furtner and Rauthmann, 2011) and these strategies have a unique and strong relationship with job performance (Furtner et al., 2015).

Besides the positive effects of self-leadership on work engagement, there is also some evidence for the positive effects of self-leadership on outcomes related to mental and physical health. Lucke and Furtner (2015) find that training of self-leadership for soldiers contributed to their physical and mental performance. And Unsworth and Mason (2012) find that a self-leadership intervention helps to reduce work related strain while self-efficacy and positive affect increased (Unsworth and Mason, 2012).

### The Mediating Role of Self-Leadership

We assume that self-leadership mediates the relationship between job autonomy and work engagement and health, respectively, in three different ways. First, job autonomy encourages healthcare workers to take up responsibility and act on the basis of their own professional insights (Hall, 1968; Hackman and Oldman, 1976). SDT explains that the experience of freedom within a job changes the motivation from controlled to autonomous motivation (Gagné and Deci, 2005). The reduction of external control and, thus, the improvement of job autonomy stimulate actual self-leadership behavior. The actual autonomous functioning satisfies the basic need for autonomy and, therefore, contributes to work engagement and health. Second, job autonomy facilitates employees to determine their own way of working and to bring this in line with personal preferences (Deci and Ryan, 2000). The absence of external control allows healthcare workers to complete their tasks in their own favorite way and also to concentrate their mental focus on the naturally rewarding aspects of the job rather than on the things that must be done. Because natural rewards strategies aim to improve intrinsic motivation and reduce the focus on external behavior regulations, we expect an increase in work engagement and health (Ryan and Deci, 2008).

Third, job autonomy enables healthcare workers to take charge of job demands and the achievement of work-related goals (Bakker and Demerouti, 2007). The job demands of healthcare workers can sometimes be challenging, difficult, or boring though the work still needs to be done. Experiencing job autonomy encourages employees to take charge of organizing job demands by using behavioral and cognitive self-leadership strategies (Müller and Niessen, 2019). By using these strategies, healthcare workers experience more control in their work, leading to more work engagement and health even in a highly demanding work environment (Lovelace et al., 2007).

Based on the arguments above, we propose that the facilitation of job autonomy encourages healthcare professionals to take the lead, which explains the positive effects of job autonomy on work engagement and health. We hypothesize the following:

Hypothesis 2: Self-leadership behavior mediates the relationship between job autonomy and (a) work engagement and (b) general health of healthcare workers.

Hypothesis 3: Self-leadership natural rewards strategies mediate the relationship between job autonomy and (a) work engagement and (b) general health of healthcare workers.

Hypothesis 4: Self-leadership cognitive and behavioral strategies mediate the relationship between job autonomy and (a) work engagement and (b) general health of healthcare workers.

## Methods

### Sample and Procedure

Data was collected from two samples from organizations within the Dutch healthcare industry. The Dutch healthcare industry (including the welfare sector) is one of the largest employers in the Netherlands. Almost one in six working people (more than 1.2 million people) work in healthcare, including hospitals, nursing homes, disability care, psychiatric care, home care, and youth care. The majority (more than 70%) of these employees are women. Employees in this sector are, on average, slightly older than in the rest of the Dutch labor market (CBS, 2019).

The first sample (Organization A) was collected within three divisions (*N* = 722) of an organization for disabled and/or psychiatric clients. The second sample was collected among the full working population of a nursing home (*N* = 377) (Organization B). The first organization uses a management strategy that stimulates self-leadership. Employees work in self-management teams although managers are still responsible. Within this organization employees are strongly encouraged to take ownership of work-related problems and solve these problems independently. The second organization is a more traditionally organized nursing home in which every team has its own manager, and self-leadership is not actively stimulated.

Employees were invited by email to fill in an online questionnaire, and a paper version of the questionnaire was also available. Respondents were ensured of anonymity, and as an incentive, they could fill in their email address if they appreciated individual feedback on their score. Data collection resulted in a response-rate of 31% (*N* = 224) in Organization A and 30% (*N* = 113) in Organization B. Respondents were social workers, nurses, and paramedical staff members. Only 1.5% (*N* = 5) had a management role. In Organization A, 69% (*N* = 155) of the respondents were female, and in Organization B, this percentage was about 86% (*N* = 93). The uneven distribution of males and females in our sample is in line with the overall distribution of gender across healthcare organizations in the Netherlands. The average age of respondents was similar across both organizations (Organization A: 41.5 and Organization B: 40.1). Finally, 9% of the respondents in Organization A completed primary/secondary school, 36% completed vocational training, and 52% completed a college degree. In Organization B, 26% completed primary/secondary school, 54% completed vocational training, and 20% completed a college degree. The average age of the merged sample was 41 years (*SD* = 12.8) and 75% was female. And 15% completed primary/secondary school, 42% completed vocational training, and 41% completed a college degree.

### Measurement Instruments

#### Job Autonomy

In line with suggestions from self-leadership theory (Stewart et al., 2011), job autonomy was measured with the nine-item scale for job autonomy developed by Morgeson and Humphrey (2006). This scale captures a broad range of aspects concerning job autonomy, which is within self-leadership theory theorized to be representative of the degree to which employees experience autonomy within their job. Three dimensions of job autonomy are included, which are decision-making autonomy, work-scheduling autonomy, and work-method autonomy. These items refer to decision-making autonomy (three items, e.g., “The job allows me to make a lot of decisions on my own”), work-scheduling autonomy (three items, e.g., “The job allows me to decide on the order in which things are done on the job”), and work-method autonomy (three items, e.g., “The job allows me to make decisions about what methods I use to complete my work”). The full nine-item scale shows sufficient reliability (α = 0.95). Employees responded on a five-point response scale ranging from strongly disagree (1) to strongly agree (5).

#### Self-Leadership

For getting insight into the self-leadership process, we chose three different perspectives on self-leadership. Self-leadership behavior (SLB) is assumed to represent the actual autonomous behavior of employees (Yun et al., 2006). Following the suggestions by Houghton et al. (2012), we used both the abbreviated self-leadership questionnaire (ASLQ) (Houghton et al., 2012) for getting insight into the cognitive and behavioral strategies (SLS) and the natural rewards subscale (Houghton and Neck, 2002) as these might separately influence outcomes related to motivation.

*SLB* was measured by the six-item self-leadership measure as used by Yun et al. (2006). Example items of this scale are “I solve problems when they pop up, without always getting my supervisor’s stamp of approval,” “I take initiatives on my own,” and “I assume responsibilities on my own.” The reliability of the self-leadership behavior scale was good (α = 0.90). Employees responded on a five-point response scale ranging from strongly disagree (1) to strongly agree (5).

*Self-leadership natural rewards strategies* were measured with the five-item natural self-rewards strategies scale (Houghton and Neck, 2002). Sample items are “I seek out activities in my work that I enjoy doing” and “I focus my thinking on the pleasant rather than the unpleasant aspects of my job activities.” The measure showed sufficient reliability (α = 0.85). Employees responded on a five-point response scale ranging from strongly disagree (1) to strongly agree (5).

*SLS* were measured by the ASLQ (Houghton et al., 2012), which represents three subfactors: “behavior awareness and volition” (goal setting and self-observation), “task motivation” (mental imagery and self-reward), and “constructive cognition” (positive self-talk and evaluation of beliefs and assumptions). A sample item for behavioral awareness and volition is “I establish specific goals for my own performance.” A sample item for task motivation is “I visualize myself successfully performing a task before I do it.” A sample item for constructive cognition is “I try to mentally evaluate the accuracy of my own beliefs about situations I am having problems with.” The ASLQ showed good reliability (α = 0.88). Employees responded on a five-point response scale ranging from strongly disagree (1) to strongly agree (5).

*Work engagement* was measured using the nine-item Utrecht Work Engagement Scale (Schaufeli et al., 2006), which consists of three subscales: vigor, dedication, and absorption. A sample item is “At my work, I feel strong and vigorous.” Employees responded on a seven-point response scale ranging from never (1) to always (7). The measure showed good reliability (α = 0.93).

*General health* was measured with a single item “How would you rate your general health at this moment?” (Hooftman et al., 2017). Respondents answered on a six-point Likert scale ranging from very bad (1) to very well (6).

#### Control Variables

We controlled for age, gender, organization, and educational level because prior research pointed out that these influence self-leadership (Ugurluoglu et al., 2015).

### Analyses

We tested our hypotheses using a series of regressions in Mplus (Muthén and Muthén, 2017). First, we tested Hypothesis 1 by regressing the two dependent variables, work engagement and health, on job autonomy, including our control variables. To test Hypotheses 2, 3, and 4, we first regressed our mediators (self-leadership behavior, self-leadership cognitive and behavioral strategies, and self-leadership natural rewards strategies) on job autonomy. In the second step, we regressed the dependent variables, work engagement and health, on the mediators and job autonomy. To assess the significance of the indirect effects proposed on Hypotheses 2, 3, and 4, we used bootstrapping with 5000 resamples. Because we are not interested in comparing effect sizes, we report the unstandardized beta weights.

## Results

### Measurement Model

Before we tested our hypotheses, we examined the discriminant validity of our measurement model. We used a CFA to test different models using different combinations of our main study variables. Because our measures of job autonomy (decision-making autonomy, work-scheduling autonomy, and work-method autonomy), self-leadership strategies (behavior awareness and volition, task motivation, and constructive cognition), and work engagement (vigor, dedication, and absorption) consist of multiple dimensions, we model these constructs as second order factors with underlying first order factors. First, we tested a model in which all variables (job autonomy, self-leadership behavior, self-leadership cognitive and behavioral strategies, natural rewards, and work engagement) load on one single factor [χ^2^(665) = 5710.37, *p* < 0.001, RMSEA = 0.15, CFI = 0.38, TLI = 0.34]. Second, we tested a three-factor model in which all self-leadership-variables load on one factor [χ^2^(662) = 3300.44, *p* < 0.001, RMSEA = 0.11, CFI = 0.67, TLI = 0.67]. Next, we tested a five-factor model in which all variables load on five separate factors with the underlying dimensions of job autonomy, self-leadership strategies, and work engagement loading on second order factors [χ^2^(646) = 1321.83, *p* < 0.001, RMSEA = 0.056, CFI = 0.92, TLI = 0.91]. Finally, we also tested an 11-factor model without second-order factors in which each subdimension was considered a separate construct [χ^2^(610) = 1227.18, *p* < 0.001, RMSEA = 0.055, CFI = 0.92, TLI = 0.91]. The 11-factor model shows a better fit compared to the five-factor model with second order factors [Δχ^2^ = 95(36), *p* < 0.001]. However, we chose the more parsimonious five-factor model when testing the hypotheses because the second order constructs each show a high level of reliability and because the other fit indices are highly equal across both models.

### Hypotheses Testing

Table 1 shows the means, standard deviations, and correlations of the variables used in this study. Table 2 shows the results of the regressions used to test the hypotheses.

**TABLE 1:** Correlations, Means, and *SD*s of main variables (*N* = 337).

		Mean	*SD*	1	2	3	4	5	6	7	8	9
1	Work engagement	3.87	1.06	1								
2	General health	4.16	1.13	0.25***	1							
3	Job autonomy	3.29	0.75	0.26***	0.16**	1						
4	SLB	3.89	0.67	0.19**	0.12*	0.44***	1					
5	NR	3.67	0.59	0.54***	0.28***	0.36***	0.33***	1				
6	SLS	3.21	0.64	0.32***	0.04	0.21***	0.29***	0.41***	1			
7	Organization^a^	0.34	0.47	–0.03	0.02	−0.24***	−0.21***	–0.06	−0.12*	1		
8	Age	40.9	12.8	0.15*	–0.08	0.09	0.06	0.13*	0.05	–0.05	1	
9	Gender^b^	0.25	0.43	–0.09	0.03	0.04	–0.09	–0.05	–0.09	−0.18**	0.16**	1
10	Educational level^c^	6.90	1.66	–0.01	0.14**	0.15**	0.23***	0.02	0.07	−0.27***	−0.19**	0.12*

*p < 0.05; **p < 0.01; ***p < 0.001; SLB, Self-leadership behavior; NR, Natural rewards strategies; SLS, Self-leadership cognitive and behavioral strategies. ^*a*^0 = Organization A. ^*b*^0 = female. ^*c*^1–5 = primary/secondary school, 6–7 = vocational training, 8–9 = college degree.

**TABLE 2:** Regressions (*N* = 337).

	SLB	NR	SLS	Work engagement		Health	
				Step 1	Step 2	Step 1	Step 2
Intercept	2.46(0.35)***	2.52(0.31)***	2.60(0.30)***	2.48(0.64)***	2.49(0.64)***	2.70(0.55)***	1.77(0.68)**
**Control variables**							
Organization^a^	−0.10(0.09)	0.06 (0.08)	−0.11(0.08)	0.01 (0.02)	0.01 (0.13)	0.12 (0.14)	0.07 (0.13)
Age	0.00 (0.00)	0.00 (0.00)	0.00 (0.00)	0.01(0.00)*	0.01 (0.01)	0.00 (0.01)	−0.01(0.01)
Gender^b^	−0.21(0.08)*	−0.04(0.08)	−0.14(0.08)^†^	−0.23(0.16)	−0.17(0.13)	0.08 (0.14)	0.09 (0.14)
Educational level^c^	0.09(0.03)**	−0.01(0.03)	0.03 (0.02)	−0.02(0.05)	−0.02(0.04)	0.09(0.04)*	0.09(0.04)*
**Independent variables**							
Job autonomy	0.32(0.06)***	0.30(0.05)***	0.14(0.05)**	0.39(0.09)***	0.09 (0.09)	0.20(0.09)*	0.04 (0.09)
SLB					−0.02(0.12)		0.10 (0.11)
NR					0.86(0.11)***		0.56(0.12)***
SLS					0.27(0.12)*		−0.27(0.12)*
*R*^2^	0.25	0.16	0.08	0.10	0.32	0.05	0.16

^†^p < 0.10; *p < 0.05; **p < 0.01; ***p < 0.001; SLB, Self-leadership behavior; NR, Natural rewards strategies; SLS, Self-leadership cognitive and behavioral strategies; Step 1, direct effect; Step 2, mediation effect. ^*a*^0 = Organization A. ^*b*^0 = female. ^*c*^1–5 = primary/secondary school, 6–7 = vocational training, 8–9 = college degree.

Hypothesis 1 predicted that job autonomy is positively associated with (a) work engagement and (b) general health of healthcare workers.

The results show that job autonomy is positively associated with both work engagement [*B* = 0.39(0.09), *p* < 0.001] and general health [*B* = 0.20(0.09), *p* < 0.05], which confirms Hypothesis 1.

Hypothesis 2 predicts that self-leadership behavior mediates the relationship between job autonomy and (a) work engagement and (b) general health. The results in Table 2 show that job autonomy is positively related to self-leadership behavior [*B* = 0.32(0.06), *p* < 0.001], but self-leadership behavior is not associated with work engagement [*B* = -0.02(0.12), *p* = ns] and general health [*B* = 0.10(0.11), *p* = ns], which rejects Hypothesis 2. Hypothesis 3 proposes that natural rewards strategies mediate between job autonomy and work engagement and health, respectively. We found that job autonomy is positively related to natural rewards [*B* = 0.30(0.05), *p* < 0.001], and natural rewards is also associated with work engagement [*B* = 0.86(0.11), *p* < 0.001] and general health [*B* = 0.56(0.12), *p* < 0.001]. An analysis of the indirect effect shows that the associations between job autonomy and work engagement [*B* = 0.26(0.05), *p* < 0.001, CI95% = 0.17;0.37] and general health [*B* = 0.17(0.05), *p* < 0.001, CI95% = 0.09;0.28] via natural rewards is significant, which accepts Hypothesis 3. Finally, Hypothesis 4 proposed that cognitive and behavioral self-leadership strategies mediate between job autonomy and work engagement and health, respectively. The results in Table 2 show that job autonomy is positively related to self-leadership strategies [*B* = 0.14(0.05), *p* < 0.01], and self-leadership strategies are also positively associated with work engagement [*B* = 0.27(0.12), *p* < 0.05] and negatively with general health [*B* = -0.27(0.12), *p* < 0.05]. An analysis of the indirect effect of cognitive and behavioral self-leadership strategies shows that the associations between job autonomy and work engagement [*B* = 0.04(0.02), *p* < 0.10, CI95% = 0.01;0.09] and general health [*B* = –0.04(0.02), *p* < 0.10, CI 95% = -0.09; -0.01] are marginally significant with small effect sizes. To summarize, the results from testing the mediating role of self-leadership behavior (H2), self-leadership natural rewards strategies (H3), and self-leadership cognitive and behavioral strategies (H4), we conclude that only Hypothesis 3 was fully confirmed. Furthermore, there is marginal support for Hypothesis 4 regarding the mediation effect of behavior and cognitive strategies although the effect size is small.

## Discussion

Job autonomy is broadly recognized to be one of the important job design measures for improving the willingness and ability of healthcare professionals to continue working within their industry (Cicolini et al., 2014). Building on the job demand control model by Karasek (1979), it is assumed that, if healthcare workers are facilitated with more autonomy in their work, they will be able to handle the high job demands better (Laschinger et al., 2001). According to SDT, this might be explained by the facilitation of autonomy in the social context as this is assumed to satisfy the basic psychological need for autonomy (Van den Broeck et al., 2008; Deci et al., 2017). Indeed, the present study confirms that job autonomy is positively associated with work engagement and general health. However, we also find that self-leadership (Stewart et al., 2011) explained the relationship between job autonomy and work engagement and health, respectively. Specifically, the use of natural rewards strategies fully mediates both relationships. Besides, the mediating effect of cognitive and behavioral self-influencing strategies is marginally significant though with a small effect size. Surprisingly, the cognitive and behavioral strategies are positively associated with work engagement but negatively with general health. Actual autonomous self-leadership behavior has no role in the relationship between job autonomy and work engagement and health.

### Implications for Theory

#### Job Autonomy, Self-Leadership, Work Engagement, and Health

SDT assumes that the facilitation of autonomy in this context allows employees to fully endorse what they do and, therefore, positively contributes to motivation and health. Interestingly, in the present study, autonomous self-leadership behavior, which explicitly represents the actual autonomous work behavior, does not explain the relationship between job autonomy and work engagement and health. On the basis of the present study, we propose that the theorized impact of job autonomy on the motivational process (Gagné and Deci, 2005) requires competencies in self-leadership. Specifically, natural rewards strategies and, marginally, cognitive and behavioral strategies explain the relationship between job autonomy and work engagement and health, respectively.

However, many job types, such as those of nurses and social workers, are not facilitated with full autonomy as there are numerous procedures and instructions that need to be followed. Therefore, the original intrinsic motivation can easily be thwarted by job tasks that simply must be done, resulting in controlled regulations for motivation (Gagné and Deci, 2005). Self-leadership theory assumes that people can still self-influence their motivation and performance (Stewart et al., 2019). Indeed, the present study shows that people can influence their own motivation and health by using natural rewards strategies. Natural rewards strategies represent changing both the mental focus toward positive, naturally rewarding aspects of a job and also the behaviors with the aim to make a job more intrinsically motivating. By practicing natural rewards strategies, healthcare professionals might alter the motivation from what must be done to what one is willing to do. Moreover, it is confirmed that behavioral and cognitive strategies influence work engagement although they also have a negative association with general health. This trend is in line with Zeijen et al. (2018), who find that, specifically, goal setting and self-punishment thoughts are associated with workaholism, and self-observation and goal setting are also positively associated with work engagement. Workaholism reflects the tendency to work excessively hard and being obsessed with work (Schaufeli et al., 2008). Within SDT, it is found that workaholism has a negative influence on health, which is explained by the controlled regulation of motivation (Van den Broeck et al., 2011). SDT assumes that goals are only beneficial for intrinsic motivation if these are aligned with personal values (Sheldon and Elliot, 1999; Deci and Ryan, 2000). It is proposed that goal striving only has long-term and positive effects on well-being if the goals are in concordance with personal values and needs. Although self-leadership theory also theorizes that behavior intentions that are based on autonomy give high-quality outcomes related to general functioning (Manz, 2015), it does not explicate goal-setting strategies into intrinsic and extrinsic goals. By referring to Latham and Locke (1991) as well as to Bandura (1977), self-leadership theory assumes that goal setting in general contributes to self-motivation for the actual goal achievement (Neck and Houghton, 2006). However, on the basis of the present study and on insights by SDT (Ryan and Deci, 2017), we propose to make a difference between extrinsically and intrinsically regulated self-leadership strategies. If the self-leadership strategies are fully endorsed by the individual, they are based on autonomy. As a result, they might contribute to both work engagement and health. However, if behavioral or cognitive strategies are based on controlled regulations for behavior, this might negatively influence the health of the employees (Weinstein and Ryan, 2011). For instance, Zeijen et al. (2018) includes self-punishment within the study. Self-punishment thoughts are highly critical and self-controlling and, therefore, are assumed to reflect introjected motivation as theorized by SDT (Gagné and Deci, 2005). Introjected regulation refers to intrapersonal processes with the aim to control personal behavior in order to build better self-esteem. Self-leadership scholars argue that these types of strategies can be detrimental for motivation and performance and, therefore, should be avoided (Neck and Houghton, 2006). In contrast, the cognitive natural rewards strategies seem to be better strategies as the present study confirms their positive impact on both work engagement and health.

Notably, both SDT (Gagné and Deci, 2005) and self-leadership theory (Stewart et al., 2011) use a continuum for explaining the regulation of motivation. SDT explains the motivational process along a continuum from controlled to autonomously regulated motivation. Self-leadership theory explains the self-influencing process from low control to high control over the what, why, and how of the job. We propose that the self-leadership continuum might be extended by more explicitly using insights from SDT. Future research should include the full-range motivational continuum as explained by SDT (Gagné and Deci, 2005) and subsequently test how the different self-leadership strategies can influence the motivational process in such a way that motivation becomes more autonomously regulated while controlled motivation reduces.

#### The Contribution of Self-Leadership for Healthcare Workers

The present study found evidence for the relevance of self-leadership regarding work engagement and health of healthcare professionals. Although the healthcare literature assumes that increasing job autonomy is important for the well-being of employees, the present study shows that an individual’s self-leadership should be taken into account. If healthcare workers are able to take the lead, they are able to make better use of job autonomy. Whereas the two organizations within our sample differed in their management strategy concerning the level of autonomy, this did not influence our results. This is in line with findings by Presseau et al. (2014). It seems that, specifically, the individual’s self-leadership explains the outcomes of job autonomy on work engagement and health. We propose that, if healthcare workers experience job autonomy, they still might have the idea that they do their activities on a basis of what *must* be done. Kubicek et al. (2014) even find that too much job autonomy can have detrimental effects on the health and work engagement of healthcare workers. Probably, the increased responsibility that comes along with the increased job autonomy might feed the controlled motivation as one is insecure concerning the actual autonomous functioning. However, the self-leadership literature assumes that, through self-leadership, people will increase the self-efficacy concerning their performance (Prussia et al., 1998), and moreover, self-efficacy will buffer the negative effects of high-strain work environments (Lovelace et al., 2007; Unsworth and Mason, 2012). If we follow that line, in order to increase the job autonomy of healthcare professionals, attention needs to be paid to the training of self-leadership, especially if they are not sufficiently able to take the lead.

### Limitations

This study has several strengths, including the single focus on healthcare organizations and the multidimensional measurement of self-leadership. However, this study also has a number of limitations. First, causality cannot be unequivocally determined given the cross-sectional nature of the data. However, theoretical justification and logical arguments have been provided in support of the proposed directionality of the relationships examined. Nevertheless, it is also theorized that engaged employees are more proactive (Bakker et al., 2014), which might result in more initiative concerning the achievement of personal goals and the satisfaction of psychological needs. The job crafting literature (e.g., Demerouti et al., 2015) has already shown that people can also proactively organize more job resources, such as job autonomy for themselves, which consequently functions as nutriment for the work engagement. Furthermore, the broaden-and-build theory proposes a positive gain spiral between thought, actions, and emotions (Fredrickson, 2001). If self-leadership leads to positive affect this functions as positive feedback and, as such, further encourages the use of self-leadership. This might also explain the high correlation between natural rewards strategies and the work engagement in our study. The actual strategies might directly result in work engagement, which, in turn, leads to even more use of natural rewards strategies. Future research should test our hypotheses and potential reciprocal relationships by using longitudinal designs or by using interventions that aim at increasing job autonomy and/or self-leadership.

Second, we assessed health using a self-reported single item measure. Although this measure is well established and used in a broad range of studies, future research should aim to assess health on several dimensions or use more objective measures, such as sickness or absenteeism.

A third limitation is that we did not include other job characteristics. For example, it is expected that job autonomy and self-leadership are both specifically worthwhile in the condition of high job demands (Lovelace et al., 2007). In other words, employees are less prone to use self-leadership as they might be less challenged to achieve their work-related goals. Future research should include job demands, such as workload, as moderators to the association between job autonomy and self-leadership to further understand the conditions under which self-leadership mediates the associations between job autonomy and employee outcomes.

Fourth, the response rate was, at 30 and 31%, respectively, rather low, presumably caused by the survey participation being voluntary, which might have led to non-response bias (Groves and Peytcheva, 2008). Smith (2009) was able to test this assumption with a double sample among nurses and finds that, except for some demographic characteristics (sex, race, and national origin), there are no significant differences in the evaluations concerning job satisfaction and burnout. Moreover, Rindfuss et al. (2015) find that a low response rate might bias univariate relationships on the basis of differences in demographics, attitudes, and behaviors with the non-respondents, but not multivariate relationships (Rindfuss et al., 2015). Therefore, we assume that the potential bias caused by a low response rate in our sample is insignificant.

Last, the present research is focused on self-leadership and specifically on self-leadership behavior, cognitive and behavioral self-leadership strategies, and natural rewards strategies. Although these are theorized to be the basic constructs for self-leadership (Neck and Houghton, 2006), it is recognized that other self-regulation strategies also might be relevant to include in self-leadership research (Manz, 2015). For instance, Weigl et al. (2014) investigated the mediating role of the action self-regulation strategies as theorized by the selection optimization compensation model (SOC, Moghimi et al., 2017). It was confirmed that the relation between job autonomy and work engagement is mediated by the SOC strategies (Weigl et al., 2014). This might be explained by the autonomous character of the goal selection. Furthermore, both within SDT and self-leadership theory, the role of mindfulness is considered as a worthwhile cognitive strategy (e.g., Weinstein and Ryan, 2011; Sampl et al., 2017). Weinstein and Ryan (2011) assume that mindfulness encourages autonomous motivation and facilitates stress resilience. Therefore, we suggest extending the research focus on other self-regulating strategies, in which we specifically recommend considering the role of autonomous motivation in the self-regulating process.

### Implications for Practice

The workload in the healthcare sector is high, and this leads to high rates of absenteeism, unfulfilled vacancies, and voluntary turnover with the effect of a further increasing workload. This has put the healthcare sector in a vicious circle of problems. Only when healthcare institutions manage to keep the back door closed and retain their staff for healthcare can the vicious circle be broken. Current research shows that there is a way for healthcare institutions to close the back door and keep their staff happy and healthy. This study finds that, when employees experience job autonomy and use naturally rewarding self-leadership strategies, they increase their work engagement and health. In the end, the patients benefit from effective self-leading healthcare professionals. Engaged and healthy employees do all they can to deliver the best possible service to their clients. Kayral and Dülger (2019) find that, if healthcare professionals are capable of taking the lead, this is associated with positive outcomes related to organizational goals, such as patient safety and efficiency. Besides, healthcare workers who are able to take the lead might inspire their clients to take the lead in their health as well. Recent research shows that patients, such as those recovering from cancer surgery, benefit from self-leadership skills for continuing their rehabilitation exercises (Lee et al., 2020).

We, therefore, advise healthcare organizations to give more job autonomy to their employees and to encourage employees to work in an autonomous and self-responsible way and use natural rewards strategies. Natural rewards strategies stand for the strategy to surround oneself with objects and people that uncover your own desirable behaviors. It is specifically this ability for natural rewards strategies that will help healthcare workers to self-influence both their work engagement and health.

Employers can learn from the results of our study that both job design measures, initiated by the employer, and self-influencing strategies of the employees can improve health and work engagement. Although practicing self-leadership is a specifically personal resource to self-influence the motivation and ability to work, employers can help to improve skills for self-leadership by offering self-leadership training. It appears that healthcare professionals can develop self-leadership and that training self-leadership contributes to work engagement and performance (Van Dorssen-Boog et al., submitted) and to proactive stress coping and increasing self-efficacy (Unsworth and Mason, 2012).

## Data Availability Statement

The datasets generated for this study are available on request to the corresponding author.

## Ethics Statement

Ethical review and approval was not required for the study on human participants in accordance with the local legislation and institutional requirements. The patients/participants provided their written informed consent to participate in this study.

## Author Contributions

PD-B was main author of the article and collected the data. JJ specifically focused on the analysis of the data and on reviewing the manuscript. MV contributed to the study design. TV contributed as reviewer to the manuscript as a whole. All authors contributed to the article and approved the submitted version.

## Conflict of Interest

PD-B was employed by Intrinzis. TV was employed by Loyalis Knowledge & Consult. The remaining authors declare that the research was conducted in the absence of any commercial or financial relationships that could be construed as a potential conflict of interest.
